# Septic cardiomyopathy: a rare and life-threatening complication of small intestinal perforation: A case report

**DOI:** 10.1097/MD.0000000000049101

**Published:** 2026-06-05

**Authors:** FanJing Wang, Run Ma, YuanJia Luo, Dan Ma, JianFeng Xiao

**Affiliations:** a Department of Cardiovascular Intensive Care Unit, Northeast Yunnan Central Hospital, Zhaotong, Yunnan Province, China.

**Keywords:** septic cardiomyopathy, septic shock, small intestinal perforation

## Abstract

**Rationale:**

Septic cardiomyopathy (SCM) is a severe and often reversible complication of sepsis but carries a high mortality rate when it progresses to cardiogenic shock. Differentiating SCM from acute coronary syndrome in septic patients is a major clinical challenge. This report details a case of SCM secondary to small intestinal perforation, highlighting both its life-threatening presentation and successful management.

**Patient concerns:**

A 62-year-old male presented with acute abdominal pain and was found to have a small intestinal perforation with peritonitis. Emergency surgery was performed. Postoperatively, the patient developed profound septic and cardiogenic shock, with ST-segment elevation on the electrocardiogram mimicking an acute anterior myocardial infarction.

**Diagnoses:**

SCM, septic shock, and cardiogenic shock were diagnosed based on the postsurgical sepsis, ST-segment elevation, significantly elevated troponin levels, and a transthoracic echocardiogram showing a severely reduced left ventricular ejection fraction of 38% with diffuse hypokinesis.

**Interventions:**

A multimodal strategy was employed, including surgical source control, high-dose vasopressor support, intra-aortic balloon pump insertion, continuous renal replacement therapy to modulate the cytokine storm, and inotropic support with levosimendan.

**Outcomes:**

The patient’s hemodynamics stabilized, and cardiac function showed significant recovery, with left ventricular ejection fraction improving to 65%. The ST-segment changes resolved. He was successfully weaned from all supports and discharged.

**Lessons:**

This case highlights the diagnostic pitfall of SCM mimicking ST-segment elevation myocardial infarction. It demonstrates that early recognition, definitive source control, and a comprehensive, multimodal support strategy are critical for a favorable outcome, even in life-threatening SCM.

## 1. Introduction

Sepsis is defined as life-threatening organ dysfunction caused by a dysregulated host response to infection, and it is a disease with a high mortality rate.^[1]^ According to the latest Global Burden of Disease data, there were an estimated 48.9 million cases of sepsis and 11 million sepsis-related deaths worldwide in 2017, accounting for 19% of all global deaths.^[2]^ Septic cardiomyopathy (SCM) is a severe complication of transient cardiac dysfunction caused by systemic infection, associated with a high short-term mortality rate. Among various complications, SCM is a common and serious condition characterized by reversible myocardial dysfunction, manifesting as impaired ventricular systolic and/or diastolic function.^[3]^ When SCM is accompanied by septic shock, the prognosis of patients significantly worsens, with mortality rates as high as 70% to 90%.^[4]^ The diagnosis of SCM presents numerous challenges, primarily due to the current lack of a clear objective definition, as well as difficulties in differentiating it from other acute cardiac dysfunctions such as acute coronary syndrome (ACS), especially in the context of severe sepsis.^[5]^ Its pathogenesis is multifactorial, involving a complex interplay of inflammatory mediators, mitochondrial dysfunction, and metabolic disturbances, which ultimately lead to depressed cardiac function.^[6]^

The clinical trajectory from a localized infection to fulminant sepsis and subsequent SCM can be rapid and catastrophic. This case report holds significant clinical value as it meticulously documents the complete and swift progression from a common surgical emergency – small intestinal perforation – to severe sepsis, and then to a life-threatening presentation of SCM culminating in cardiogenic shock. This narrative underscores a critical diagnostic pitfall: the presentation with ST-segment elevation mimicking an acute anterior myocardial infarction, which necessitates urgent differentiation from a primary coronary event to guide appropriate management. The case exemplifies the paramount importance of a multidisciplinary approach, integrating emergency surgical source control, intensive care medicine, and advanced cardiac support. It provides a practical reference for the comprehensive management of such complex cases, detailing the sequential application of high-dose vasoactive support, mechanical circulatory assistance with an intra-aortic balloon pump (IABP), adjunctive therapies like continuous renal replacement therapy (CRRT) for cytokine modulation, and inotropic support with levosimendan. By illustrating a successful therapeutic strategy addressing the intertwined pathologies of septic and cardiogenic shock, this report contributes to the evolving understanding of personalized, phenotype-driven care in sepsis and its cardiovascular complications.

## 2. Case presentation

### 2.1. Ethical and consent statement

This case report was conducted in accordance with the Declaration of Helsinki. The need for ethical approval was waived by the Institutional Review Board of Northeast Yunnan Central Hospital because this is a retrospective case report of a single patient. Written informed consent was obtained from the patient for publication of this report and any accompanying images.

### 2.2. Patient information

**Figure 1.**
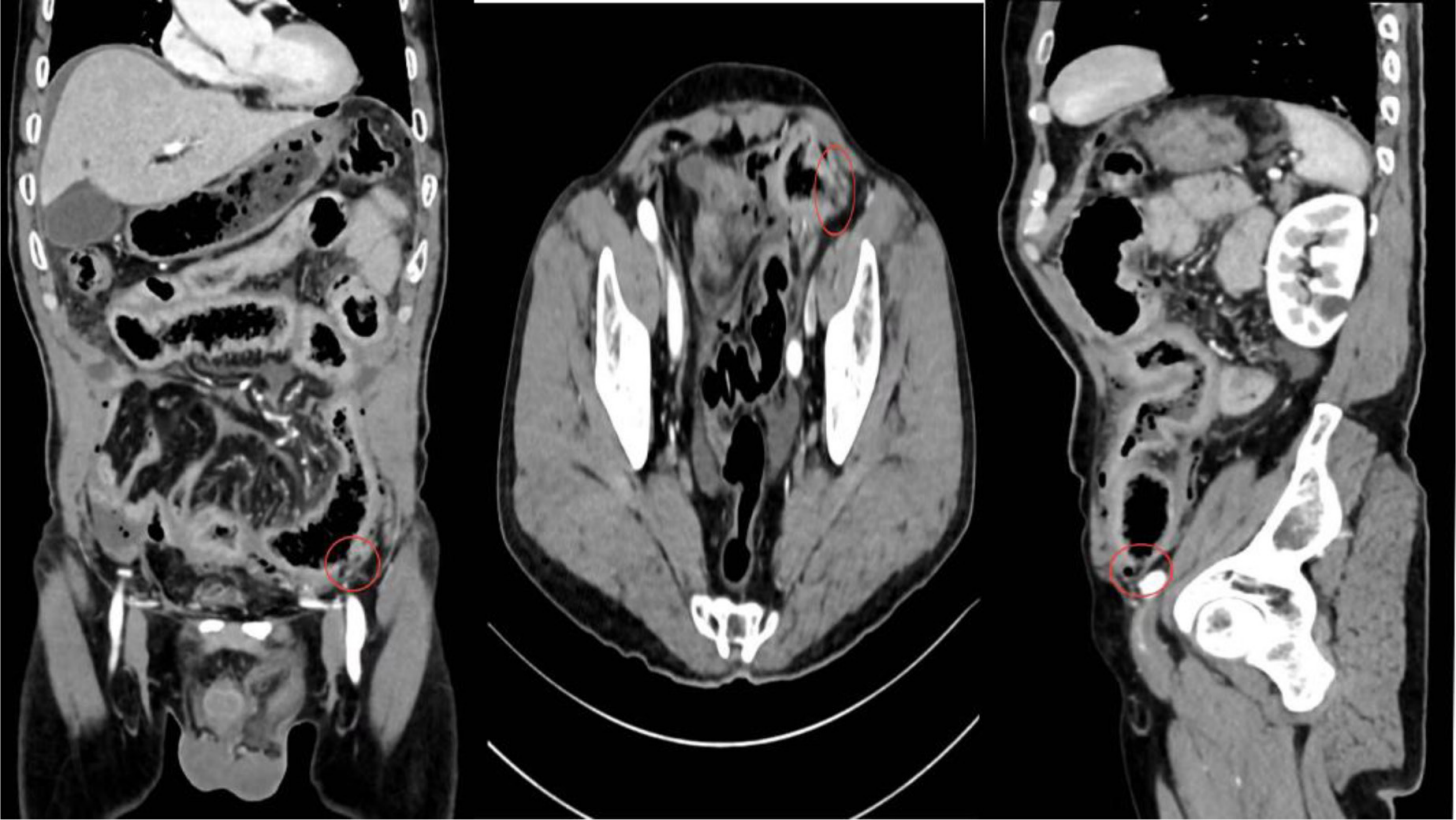
Preoperative abdominal CT showed the site of small bowel perforation. CT = computed tomography.

A 62-year-old male patient presented to the emergency department with a chief complaint of abdominal pain for 1 day. The pain was sudden in onset, colicky in nature, persistent, and most pronounced in the lower abdomen. He reported no associated nausea, vomiting, diarrhea, or fever. His past surgical history was significant for a right inguinal hernia repair performed 7 years prior, with no known history of gastrointestinal disease. Upon presentation, a physical examination revealed abdominal muscle rigidity and generalized tenderness. An urgent abdominal computed tomography scan demonstrated multiple foci of free intraperitoneal air, suggestive of a hollow viscus perforation, and a left inguinal hernia (Fig. 1). Preoperative assessments, including electrocardiogram (ECG), echocardiography, and infection markers, were unremarkable, and no surgical contraindications were identified, leading to the decision for emergency surgical intervention.

### 2.3. Clinical findings

During the exploratory laparotomy, a significant amount of purulent fluid was encountered within the peritoneal cavity. A 1 × 1 cm perforation was identified in the wall of the small intestine. Approximately 30 cm proximal to the ileocecal valve, extensive dilation and adhesions of the small bowel were noted, which were inseparable and resulted in luminal narrowing, consistent with intestinal obstruction. Consequently, an approximately 80 cm segment of small bowel was resected. The surgical procedures performed included repair of the small intestinal perforation, bowel resection with primary anastomosis, and tension-free repair of the left inguinal hernia. The intraoperative course was complicated by hemodynamic instability, requiring continuous infusion of norepinephrine to maintain blood pressure. The estimated blood loss was 300 mL, necessitating transfusion of 2 units of packed red blood cells and 400 mL of plasma. The total operative duration was approximately 5 hours, after which the patient was transferred to the intensive care unit (ICU) while still intubated and mechanically ventilated.

### 2.4. Diagnostic assessment

**Figure 2.**
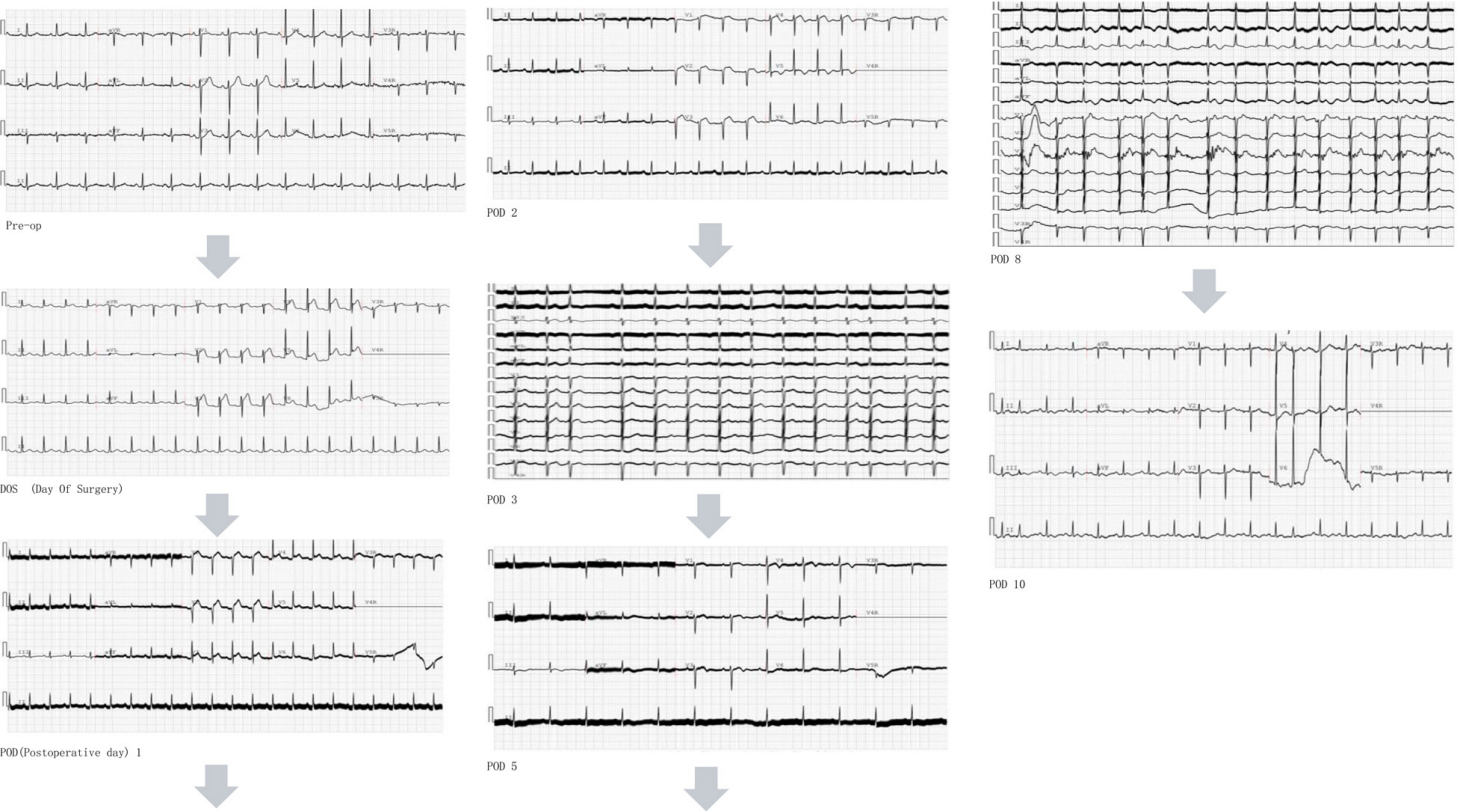
Electrocardiographic changes. Pre-op, day of surgery (DOS), POD1 to POD10.

**Figure 3.**
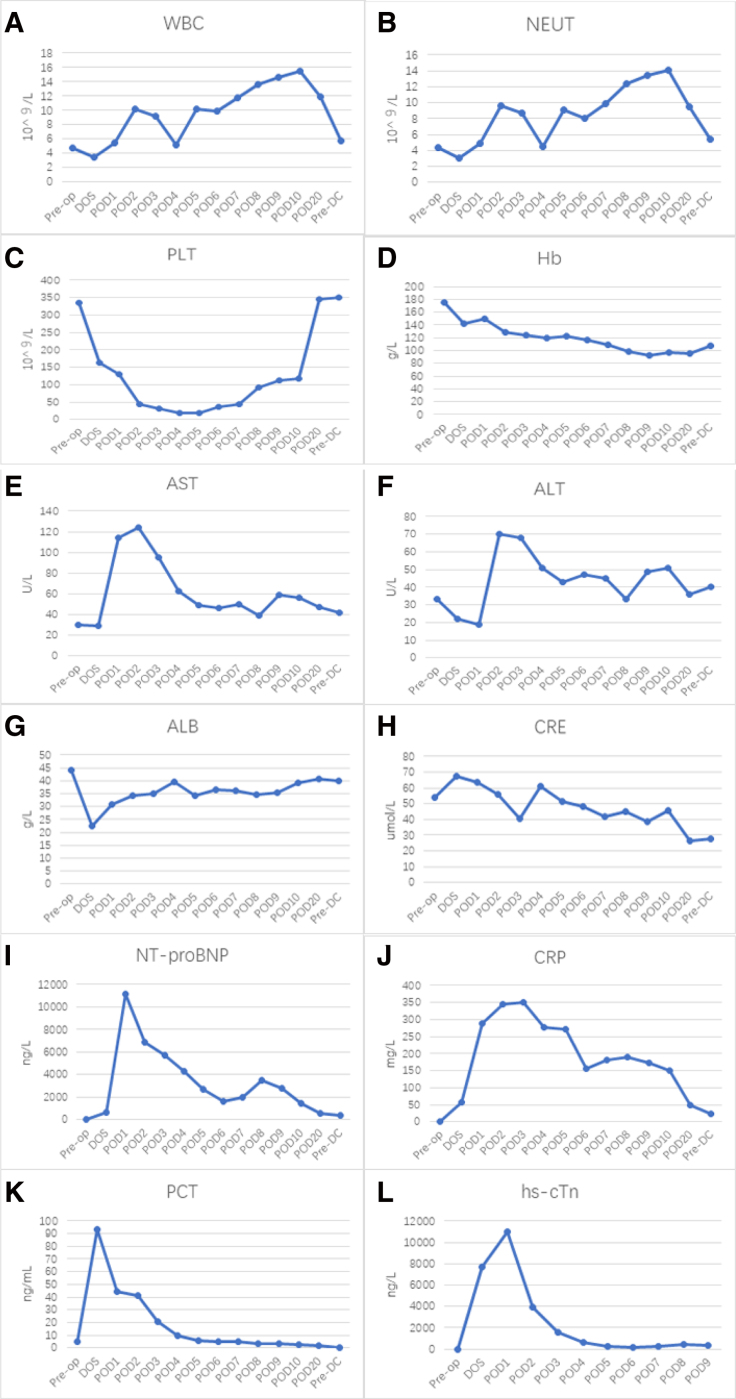
Time courses of infection-related, hepatic, renal, and cardiac function parameters (panels A–L). (A) WBC, (B) NEUT, (C) PLT, (D) Hb, (E) AST, (F) ALT, (G) ALB, (H) CRE, (I) NT-proBNP, (J) CRP, (K) PCT, (L) hs-cTn. The x-axis represents time points (pre-operation, day of surgery, and postoperative days 1–10, as indicated in the figure). ALB = albumin, ALT = alanine aminotransferase, AST = aspartate aminotransferase, CRE = creatinine, CRP = C-reactive protein, Hb = hemoglobin, hs-cTn = high-sensitivity cardiac troponin, NEUT = neutrophil count, NT-proBNP = N-terminal pro-B-type natriuretic peptide, PCT = procalcitonin, PLT = platelet count, WBC = white blood cell count.

Immediately upon ICU admission, the patient remained under deep sedation and analgesia, supported by mechanical ventilation. He developed progressive hypotension and tachycardia refractory to aggressive fluid resuscitation and escalating doses of vasopressors, including dopamine and norepinephrine. A repeat ECG revealed findings indicative of an acute extensive anterior ST-segment elevation myocardial infarction (Fig. 2). Serum troponin levels were markedly elevated. A subsequent bedside transthoracic echocardiogram demonstrated a severely reduced left ventricular ejection fraction of 38% with diffuse hypokinesis of the ventricular walls. Laboratory studies showed a significant rise in white blood cell count, C-reactive protein, and procalcitonin compared to preoperative levels (Fig. 3). Integrating these findings with the clinical context of recent sepsis due to intestinal perforation, a diagnosis of SCM, septic shock, and cardiogenic shock was established.

### 2.5. Therapeutic intervention

Given the profound and refractory circulatory collapse despite high-dose vasopressor support (norepinephrine, dopamine, and epinephrine), an IABP was emergently inserted to provide mechanical circulatory support. The patient also exhibited frequent episodes of atrial fibrillation and atrial premature contractions, which were managed with infusions of amiodarone and lidocaine. Broad-spectrum antimicrobial therapy with meropenem and vancomycin was initiated. On postoperative day 1, due to the severity of the systemic inflammatory response, CRRT was initiated to mitigate the cytokine storm. Inotropic support was augmented with the addition of levosimendan to address the low cardiac output state. The exacerbated infection resulted in significant consumption of platelets. Concurrent thrombocytopenia was treated with a transfusion of 1 therapeutic unit of platelets. Meticulous fluid balance management was maintained, aiming for a net negative fluid balance, and vasoactive drug dosages were dynamically titrated (Table 1).

**Table 1:** Vasoactive drug use and changes in cardiac function.

	Pre-op	POD1	POD2	POD3	POD4	POD5	POD6	POD7	Unit
NE	1.25	0.5	0.2	0.15	0.2	0.1	0.05	–	µg/kg/min
DA	8	7	7	10	10	6	3	–	µg/kg/min
VP	5	5	4	–	–	–	–	–	mL/h
EF	38	37	56	57	56	–	65	65	%
WM	LV apex/RV free wall hypokinesis	LV apex/RV free wall hypokinesis	Segmental LV hypokinesis	Segmental LV hypokinesis	Segmental LV hypokinesis	Segmental LV hypokinesis	Normal	Normal	–
SF	Biventricular systolic dysfunction	Biventricular systolic dysfunction	LV systolic dysfunction	Normal	Normal	Normal	Normal	Normal	–
FB	−300	+176	−220	−510	−280	−95	−48	+242	mL

DA = dopamine, EF = ejection fraction, FB = fluid balance, LV = left ventricle, NE = norepinephrine, RV = right ventricle, SF = systolic function, VP = vasopressin, WM = wall motion.

### 2.6. Follow-up and outcomes

By postoperative day 2, a downward trend in infection markers was observed, allowing for the discontinuation of CRRT. The patient developed hepatic dysfunction and hypoalbuminemia. Aggressive management was undertaken to improve liver function and correct hypoalbuminemia. Vasopressor requirements began to decrease gradually. From postoperative days 3 to 7, the patient’s clinical condition steadily improved. Infection parameters continued to decline, enabling the progressive weaning and eventual cessation of all vasoactive medications. Follow-up echocardiograms documented a recovery in left ventricular function, with an increasing left ventricular ejection fraction and improved wall motion. The ST segment on the ECG gradually resolved to normal (Fig. 2). The IABP was successfully removed on postoperative day 8. The patient was successfully extubated on postoperative day eleven. Laboratory tests revealed a continuous increase in platelet count, along with persistent declines in inflammatory markers and troponin levels. Liver function showed improvement, and although hemoglobin decreased, it remained within the normal range. The patient’s condition was considered effectively controlled (Fig. 3). After demonstrating stable vital signs and sustained clinical improvement, he was transferred to a general ward on postoperative day twelve. The patient was subsequently discharged from the hospital. Unfortunately, the patient did not return for regular follow-up.

## 3. Discussion

The present case of SCM following small intestinal perforation and peritonitis underscores a distinct infectious etiology compared to the more commonly reported sources in the literature. While numerous studies document SCM arising from pulmonary infections, such as severe community-acquired pneumonia or ventilator-associated pneumonia in critically ill patients,^[7]^ and from urinary tract infections, particularly in diabetic or elderly populations,^[8]^ this case highlights a severe intra-abdominal source. Gastrointestinal perforations, leading to polymicrobial peritonitis and sepsis, represent a less frequently emphasized but equally critical pathway to myocardial dysfunction. This aligns with observations in other severe abdominal pathologies, such as complicated appendicitis with intra-abdominal sepsis^[9]^ and necrotizing pancreatitis,^[10]^ where systemic inflammation can precipitate multi-organ failure.

Furthermore, the severity of cardiac compromise in this case, manifesting as profound cardiogenic shock with an ejection fraction of 38% necessitating IABP support, illustrates the extreme end of the SCM spectrum. This contrasts with many reported instances of SCM where myocardial depression, while significant, may not progress to refractory shock requiring advanced mechanical circulatory support.^[11]^ The rapid clinical deterioration, coupled with ST-segment elevation on the ECG mimicking acute myocardial infarction, presents a critical diagnostic challenge, emphasizing the necessity to differentiate SCM from primary coronary events in the setting of overwhelming sepsis.^[12]^ The successful outcome here, achieved through source control surgery and a multimodal support strategy including IABP, CRRT, and inotropic support with levosimendan, provides a practical framework for managing such severe presentations, which may be less comprehensively detailed in reports of SCM from more common infectious sources.^[13]^

The diagnostic challenge presented by this case, particularly the ST-segment elevation myocardial infarction-like electrocardiographic changes and elevated troponin, underscores a critical teaching point in distinguishing SM from ACS. In the context of overwhelming sepsis, such findings should prompt a high index of suspicion for SCM to avoid the diagnostic trap of initiating inappropriate antiplatelet or anticoagulant therapies for presumed ACS, which could exacerbate bleeding risks without addressing the underlying inflammatory myocardial suppression.^[14]^ The pathophysiological mechanisms linking intestinal perforation to myocardial dysfunction are multifaceted. The initial insult leads to a massive release of pathogen-associated molecular patterns and damage-associated molecular patterns, triggering a systemic inflammatory response. This cascade results in the overproduction of cytokines and nitric oxide, which directly impair cardiomyocyte contractility and calcium handling.^[15]^ Furthermore, mitochondrial dysfunction emerges as a central mechanism, characterized by disrupted oxidative phosphorylation, excessive reactive oxygen species production, and impaired energy metabolism, collectively contributing to bioenergetic failure within the myocardium.

The comprehensive therapeutic strategy employed here offers valuable insights into managing severe SCM. While source control via emergency surgery remains the cornerstone, the adjunctive use of advanced modalities was pivotal. The application of IABP provided essential hemodynamic bridging by reducing afterload and improving coronary perfusion, a crucial intervention in the setting of cardiogenic shock compounded by septic vasodilation.^[4]^ The initiation of CRRT, while primarily for renal support, likely conferred benefits through the nonspecific clearance of circulating inflammatory mediators, thereby modulating the cytokine storm driving myocardial depression.^[3]^ The selection of levosimendan as an inotrope was particularly astute, as its calcium-sensitizing action improves contractility without significantly increasing myocardial oxygen demand, and its vasodilatory properties may aid in microcirculatory perfusion, addressing another key pathophysiological component of sepsis-induced organ dysfunction.^[5]^ This multimodal approach, integrating mechanical support, blood purification, and targeted pharmacotherapy, exemplifies a sophisticated response to the complex pathophysiology of SCM and highlights the necessity of a tailored, dynamic management plan in the ICU.

This case successfully demonstrates that early recognition of the septic source, prompt surgical intervention, and rapid diagnosis of SCM are fundamental. The favorable outcome was contingent upon the timely, stepwise escalation of advanced life support, including mechanical circulatory assistance and organ replacement therapies. It reaffirms that SCM is a grave complication of sepsis with potentially catastrophic presentations. The cornerstone of management remains definitive infection source control, while early identification of cardiac involvement and the initiation of a comprehensive, multimodal treatment strategy, incorporating mechanical support when indicated, are critical for improving prognosis.

This report has inherent limitations as a single-case, retrospective description, lacking a control group for comparison. We were unable to perform more sophisticated myocardial biomarker or immunological assays to elucidate the specific mechanistic pathways involved in this patient’s myocardial depression. Consequently, the therapeutic experience described, while instructive, requires validation through larger, prospective studies to better define optimal management protocols for this severe disease phenotype.

## Acknowledgments

The authors would like to thank the patient for providing informed consent and the nursing staff of the ICU for their assistance.

## Author contributions

**Software:** FanJing Wang, Dan Ma.

**Supervision:** FanJing Wang.

**Validation:** FanJing Wang, Dan Ma.

**Writing – Original Draft:** FanJing Wang, JianFeng Xiao.

**Data Curation:** Run Ma, YuanJia Luo, JianFeng Xiao.

**Funding Acquisition:** Run Ma, YuanJia Luo.

**Investigation:** Run Ma.

**Methodology:** Run Ma.

**Project Administration:** YuanJia Luo, Dan Ma.

**Resources:** Dan Ma.

**Writing – Review & Editing:** JianFeng Xiao.
